# New perspectives with the use of noninvasive chromosome screening (NICS) in ART

**DOI:** 10.5935/1518-0557.20190071

**Published:** 2019

**Authors:** José G. Franco Jr

**Affiliations:** 1 Center for Human Reproduction Prof. Franco Jr, Ribeirão Preto, SP, Brazil

**Keywords:** NICS, noninvasive chromosome screening, PGS, PGTA, cell free DNA, blastocyst

## Abstract

After more than 20 years of use of preimplantation genetic tests for aneuploidies (PGS/PGT-A) there are still many problems related to the efficiency of this technique, most of them still without an adequate solution (Gleicher *et al*., 2018; Homer, 2019). From the clinical point of view, the benefits attributed to invasive PGT-A in the selection of euploid embryos remain controversial, especially due to the lack of scientific proof of its effectiveness in increasing live birth rates in various clinical situations, such as patients with advanced age, repeated implantation failures or recurrent miscarriages. In addition, evidence-based medicine also severely criticizes the rare randomized trials analyzing the clinical use of invasive PGT-A (Orvieto, 2016). If these criticisms were not enough, and undoubtedly one of the most important, it would be difficult to accurately assess the presence of embryonic mosaicism creating significant levels of false positive results, and worse, causing a real possibility of discarding healthy embryos. This makes the clinical application of PGT-A as a risky approach (Munné *et al*., 2017; Spinella *et al*., 2018). Another problem, not less important, would be the obligation to perform PGT-A by experienced embryologists, since otherwise the embryonic loss due to biopsy would be a frequent fact, something usually estimated below 10% but in some laboratories it may reach up to 30% of biopsied embryos (Munné, 2018). On the other hand, there are doubts about the future risks of invasive action of the usually 5-10 cell removed during biopsy for genetic diagnosis. Would there be repercussions for the health of these children? In animals, there are data suggesting that embryonic biopsies could be linked to changes in fetal neural tube or adrenal development (Wu *et al*., 2014; Zeng *et al*., 2013). Recently, Xu *et al*. (2016) described noninvasive chromosomal screening (NICS) by obtaining and sequencing free DNA dripped by embryos in the culture medium (without the need of embryo biopsy) creating a new non-aggressive and elegant perspective to preimplantation genetic diagnosis.

After more than 20 years of use of preimplantation genetic tests for aneuploidies (PGS/PGT-A) there are still many problems related to the efficiency of this technique, most of them still without an adequate solution (Gleicher *et al.*, 2018; Homer, 2019).

From the clinical point of view, the benefits attributed to invasive PGT-A in the selection of euploid embryos remain controversial, especially due to the lack of scientific proof of its effectiveness in increasing live birth rates in various clinical situations, such as patients with advanced age, repeated implantation failures or recurrent miscarriages. In addition, evidence-based medicine also severely criticizes the rare randomized trials analyzing the clinical use of invasive PGT-A (Orvieto, 2016). If these criticisms were not enough, and undoubtedly one of the most important, it would be difficult to accurately assess the presence of embryonic mosaicism creating significant levels of false positive results, and worse, causing a real possibility of discarding healthy embryos. This makes the clinical application of PGT-A as a risky approach (Munné *et al.*, 2017; Spinella *et al.*, 2018).

Another problem, not less important, would be the obligation to perform PGT-A by experienced embryologists, since otherwise the embryonic loss due to biopsy would be a frequent fact, something usually estimated below 10% but in some laboratories it may reach up to 30% of biopsied embryos (Munné, 2018).

On the other hand, there are doubts about the future risks of invasive action of the usually 5-10 cell removed during biopsy for genetic diagnosis. Would there be repercussions for the health of these children? In animals, there are data suggesting that embryonic biopsies could be linked to changes in fetal neural tube or adrenal development (Wu *et al.*, 2014; Zeng *et al.*, 2013).

Recently, Xu *et al.* (2016) described noninvasive chromosomal screening (NICS) by obtaining and sequencing free DNA dripped by embryos in the culture medium (without the need of embryo biopsy) creating a new non-aggressive and elegant perspective to preimplantation genetic diagnosis.

However, let's look at some problems that have already been resolved, and others that only the future would provide definitive answers:

1. A basic point is that the NICS does not remove embryonic cells, i.e., collecting DNA from the embryonic culture medium for genetic diagnosis does not require embryo biopsy, and consequently the facts described above immediately lost their relevance (embryo injury at the time of biopsy; need for an experienced embryologist to perform the biopsy; and finally, the possible epigenetic effects).

2. Comparative studies have recently been performed between the results of three genetic analyzes (Huang *et al.*, 2019), those reported with invasive PGT-A blastocyst biopsy versus those obtained with NICS in those same cultured blastocysts for 24 hours, and lastly, of the total DNA obtained from the blastocysts now donated for research (gold standard). False positive results were significantly less common in NICS (20%) than PGT-A (50%) when both were compared with total blastocyst screening as gold standard. On the other hand, there was 100% agreement between NICS and genetic analysis of the donated blastocyst when referring to the diagnosis of euploid embryo.

Theoretically, according to Hardy *et al*. (2003) it would not be unrealistic to estimate that these donated blastocysts would have at the time of their genetic analysis somewhere around 70 cells, representing the sum of cells of internal mass (25 cells) and trophoblasts (45 cells). Although there are disagreements as to the total number of blastocyst cells however the representativeness (36% of total) of cells of the internal mass could not be neglected. Although the origin of cell-free DNA in culture medium still being discussed, one cannot disregard the role of apoptosis in the inner cell mass, an event that might strengthen the correlation between NICS and total blastocyst genetic screening (gold standard) results, since PGT-A evaluates only trophoblastic cells.

3. Recently, several papers described the births of healthy children from euploid blastocysts selected by NICS in IVF programs and also for couples carrying genetic alterations such as Robertsonian or balanced translocations and chromosomal inversions (Xu *et al.*, 2016; Fang *et al.*, 2019; Jiao *et al.*, 2019).

4. However, the validity of NICS in clinical contexts of repeated implantation failure, recurrent miscarriage, advanced age, etc., is yet to be attested. These populations have not been described in randomized trials with or without NICS.

5. In 2019, twelve Assisted Reproduction Centers started a group called NICS-Brazil to further development of NICS. Preliminary data from 24 embryos donated to research (diagnosed with anomalies by prior PGT-A) revealed that the cell-free DNA collected from blastocyst culture medium yielded 7% false positive results and no false negative result versus total blastocyst screening (gold standard). On the other hand, when the previous results obtained by invasive PGT-A were compared with blastocyst analysis (gold standard), the incidence of false positive results from PGT-A was 39%.

6. NICS is a promising method that may immediately decrease the incidence of false positive results in preimplantation genetic screening. In addition, prevent possible losses of embryos during biopsy. 

Is NICS moving to become the preferred method to selected euploid embryos in ART? The time will give the answer.

**NICS-BRAZIL:** *Genesis, CRH São José Rio Preto, IMR-Instituto de Medicina Reprodutiva e Fetal*, Feliccità, *Fertility, Ferticlin, Fertivitro, Ceferp, Materbaby, Bios, Cegonha Medicina Reprodutiva, CRH Prof. Franco Junior.*
